# Resource polymorphism in European whitefish: Analysis of fatty acid profiles provides more detailed evidence than traditional methods alone

**DOI:** 10.1371/journal.pone.0221338

**Published:** 2019-08-20

**Authors:** Stephen M. Thomas, Martin J. Kainz, Per-Arne Amundsen, Brian Hayden, Sami J. Taipale, Kimmo K. Kahilainen

**Affiliations:** 1 Department of Fish Ecology and Evolution, EAWAG Swiss Federal Institute of Aquatic Science and Technology, Center for Ecology, Evolution and Biogeochemistry, Kastanienbaum, Switzerland; 2 WasserCluster Lunz – Inter-University Centre for Aquatic Ecosystem Research, Aquatic Lipid and Ecotoxicology Research Group (LIPTOX), Lunz am See, Austria; 3 Faculty of Biosciences, Fisheries and Economics, Department of Arctic and Marine Biology, UiT The Arctic University of Norway, Tromsø, Norway; 4 Canadian Rivers Institute, Biology Department, University of New Brunswick, Fredericton, New Brunswick, Canada; 5 Department of Biological and Environmental Science, University of Jyväskylä, Jyväskylä, Finland; 6 Inland Norway University of Applied Sciences, Department of Forestry and Wildlife Management, Koppang, Norway; Uppsala Universitet, SWEDEN

## Abstract

Resource polymorphism—whereby ancestral generalist populations give rise to several specialised morphs along a resource gradient—is common where species colonise newly formed ecosystems. This phenomenon is particularly well documented in freshwater fish populations inhabiting postglacial lakes formed at the end of the last ice age. However, knowledge on how such differential exploitation of resources across contrasting habitats might be reflected in the biochemical compositions of diverging populations is still limited, though such patterns might be expected. Here, we aimed to assess how fatty acids (FA)—an important biochemical component of animal tissues—diverged across a polymorphic complex of European whitefish (*Coregonus lavaretus*) and their closely related monomorphic specialist congener vendace (*Coregonus albula)* inhabiting a series of six subarctic lakes in northern Fennoscandia. We also explored patterns of FA composition in whitefish’s predators and invertebrate prey to assess how divergence in trophic ecology between whitefish morphs would relate to biochemical profiles of their key food web associates. Lastly, we assessed how information on trophic divergence provided by differential FA composition compared to evidence of resource polymorphism retrieved from more classical stomach content and stable isotopic (δ^13^C, δ^15^N) information. Examination of stomach contents provided high-resolution information on recently consumed prey, whereas stable isotopes indicated broad-scale patterns of benthic-pelagic resource use differentiation at different trophic levels. Linear discriminant analysis based on FA composition was substantially more successful in identifying whitefish morphs and their congener vendace as distinct groupings when compared to the other two methods. Three major FA (myristic acid, stearic acid, and eicosadienoic acid) proved particularly informative, both in delineating coregonid groups, and identifying patterns of pelagic-benthic feeding throughout the wider food web. Myristic acid (14:0) content and δ^13^C ratios in muscle tissue were positively correlated across fish taxa, and together provided the clearest segregation of fishes exploiting contrasting pelagic and benthic niches. In general, our findings highlight the potential of FA analysis for identifying resource polymorphism in animal populations where this phenomenon occurs, and suggest that this technique may provide greater resolution than more traditional methods typically used for this purpose.

## Introduction

Resource polymorphism—whereby distinct sympatric subpopulations of a species exploit contrasting resources—is a phenomenon characteristic of populations expanding into novel environments, being most commonly observed during colonisation of newly formed lakes or remote islands [1]. Such differential trophic specialization across populations of a single species may originate from plastic responses to environmental variation, adaptive evolution, or a combination of both phenotypic plasticity and genetic mechanisms [2–4]. Regardless of the specific mechanism via which it arises, divergence allowing exploitation of distinct resources typically occurs in response to increased ecological opportunity as a result of vacant niches and a lack of competitor species in newly colonised environments [2,3]. Following initial divergence, differential selection pressures linked to efficient capture of resources in contrasting habitats often results in phenotypic segregation among sub-populations [5,6]. As such adaptive morphological differences between populations accumulate, changes may ultimately become apparent in consumer tissue composition, due to differential ingestion of molecularly distinct food sources [7–9]. Among the functional compounds most likely to ultimately diverge as a result of such resource polymorphism are physiologically-essential fatty acids (hereafter, FA). These key constituents of animal tissues are derived from primary producers that support higher trophic levels across contrasting habitats [10]. As such, differences in FA composition are likely to be particularly pronounced in polymorphic lake fish, as discrete populations often develop distinct morphological adaptations allowing them to exploit prey resources derived from contrasting littoral, pelagic and profundal environments [7,8].

To explore how differences in trophic ecology within polymorphic species ultimately affect tissue composition, the ability to reliably quantify resource use across populations is crucial. Although trophic interactions within ecosystems can be detected directly via observation of feeding preferences, faecal investigation or gut content analysis [6,11–13], such methods are time consuming, often biased towards certain prey species due to variable identification success, and not generally applicable to all trophic levels (e.g. bacteria, microinvertebrates) [14]. In contrast, stable isotope analysis can provide a more readily assessed, longer-term integrator of assimilated diet [15], and this method has proven efficient for defining broad-scale food web structure in many terrestrial and aquatic ecosystems [16,17]. However, a major drawback of bulk stable isotopic analysis is the lack of detailed resolution of specific trophic interactions and retained diet sources, as isotopic data are restricted to mapping the trophic transfer of elements (e.g., C and N), which—whilst forming major constituents of consumer tissues—are not unique to specific prey types. However, analysis of the FA composition of consumer tissues may provide a useful complementary approach, helping to overcome the limitations of these two traditional methods. In contrast to the broad-scale information captured by stable isotope ratios, FA can often be used as long-term, high-resolution dietary biomarkers, with relative content in consumer tissues directly linked to assimilation of specific prey groups, even in the same major habitat [18–20]. The high-specificity of FA suggests a greater potential for detecting small-scale differences among diverging populations. Thus, taken together, analysis of consumer stomach contents, stable isotope ratio and tissue FA composition should be able to provide a robust and integrated record of the trophic ecology of a given study organism, assisting in the detection of resource polymorphism in recently diverged populations.

Aquatic ecosystems represent ideal environments in which to study the role of FA in food webs, since pelagic phytoplankton taxa produce distinct n-3 FA which are subsequently transferred and incorporated throughout the food web [20–22]. In contrast, benthic algae—the principal primary producers in littoral habitats—are an important source of n-6 FA that are trophically transferred via benthic macroinvertebrates to fish [23,24]. In addition to these polyunsaturated FA, some less abundant saturated FA (which often have low bioconversion rates by consumers) can be used as biomarkers of energy transfer from specific primary producer groups to consumers at higher trophic levels [11,25,26]. At present, whole-lake studies using FA as dietary tracers to map energy transfer pathways through contrasting food web compartments are still extremely limited. As differential selection pressures in pelagic and benthic habitats induce fish divergence, greater insights into patterns of FA transfer across these food web compartments could further strengthen our understanding of the ecological forces driving these radiations. To date, trophic divergence within fish populations has primarily been studied via stomach content analysis and stable isotope ratios, whereas few studies have used FA to evaluate differences between polymorphic populations or their wider food webs [25,27–30]. Although the outcomes of past FA studies have been variable in these ecosystems, the identification of distinct FAs associated with the pelagic-benthic resource axis would likely facilitate their growing use as trophic markers in aquatic ecology, helping disentangle energy flow pathways and the origin of diverse biomolecules in food webs.

European whitefish (*Coregonus lavaretus*) is the most widespread, and arguably most diverse species of coregonid fish; adaptive radiation and ecological speciation is common throughout the species’ distribution, with up to five distinct morphs co-occurring in a single lake [31–35]. Whitefish divergence is especially pronounced in northern Fennoscandia, where many lakes support polymorphic populations spanning the entire pelagic-benthic resource axis [33,36]. Moreover, in the most bathymetrically complex lakes in the region, whitefish morphs have genetically and ecomorphologically diverged across all three habitats, i.e. the littoral, profundal and pelagic zones [7,35–38]. The most pronounced divergence occurs in large and deep lakes with relatively equal habitat distribution and diverse fish fauna [33]. Due to the marked trophic specialisation of whitefish morphs in such food webs [7,8,12,13], large, deep subarctic lakes may provide ideal natural models in which to assess the efficacy of FA as biomarkers and the consequences of resource polymorphism for energetic and biomolecular transfer through lake food webs. Several lakes supporting polymorphic whitefish also host their pelagic-specialised monomorphic congener vendace (*C*. *albula)* [12,37,38], presenting the opportunity to assess the magnitude of differentiation occurring across whitefish morphs in comparison to this more uniform outgroup. As such, use of dietary, stable isotope and FA analyses in unison may provide a more comprehensive understanding of the trophic processes driving and maintaining resource polymorphism within these taxa.

In the present study, we focused on polymorphic whitefish populations inhabiting six subarctic lakes located in a watercourse in northern Fennoscandia. We assessed the utility of muscle tissue FA composition as a tool to delineate polymorphic populations and their supporting energy sources, and how this compared with more traditional diet and stable isotope ratio data. We hypothesised that: i) polymorphic whitefish groups previously identified via morphological and genetic differences could also be discerned based on FA composition of their muscle tissue; ii) due to their polymorphism, whitefish FA compositions would vary to a greater extent than those of monomorphic vendace; iii) coregonid FA composition would mirror habitat use, with distinct littoral, profundal and pelagic groups apparent, reflecting patterns in seen stomach contents and stable isotope ratios. As such, we would expect increasingly pelagic whitefish morphs to become more similar in FA composition to their purely pelagic congener, vendace; iv) key FA would be linked to the utilisation of major habitat types across all trophic levels, including predators and prey of whitefish. If identified, these FA could potentially act as effective biomarkers of pelagic *versus* benthic food-web compartments more generally, complementing more widely used dietary and stable isotopic approaches.

## Materials and methods

### Ethics statement

This study was performed in strict accordance with the Finnish and Norwegian legislation. Fishing rights in Finland belong to the landowner according to the Finnish Fishing Law (5§ 27.5.2011/600). Accordingly, the fishing permits were obtained from the landowner, Finnish Forest and Park Service (permits 17.2.2009; 23.5.2010, 2.2.2011, 11.2.2014). Fish were euthanized by cerebral concussion for tissue collection immediately after their capture in accordance with the Finnish Animal Conservation Law (32§9.8.2013/584). No ethical permission is required for described scientific sampling with gill nets according to the Finnish Animal Conservation Law (7§ 28.6.2013/498). For the sampling of fish from the Norwegian localities, a fishing permission is required from the fishing right owner. Accordingly, we obtained permissions for the gill net fishing in lower Pasvik from the County Authority of Finnmark (permission reference numbers: 10/00955-5) with legal authority through LOV 1992-05-15 nr 47, §13. No ethical permission is required from the Norwegian Animal Research Authority for sampling and described activities (FOR-2015-06-18-761; Norwegian Ministry of Agriculture and Food).

### Study area

We studied six lakes with polymorphic whitefish populations located in the subarctic Paatsjoki-Pasvik watercourse in northern Finland and Norway (S1 Fig). These lakes were oligotrophic (totP: 3–9 μg l^-1^, totN: 130–240 μg l^-1^), slightly humic and typically had an ice-free season lasting from late May until late October (S1 Table). All selected lakes were broadly similar across a suite of abiotic variables, contained distinct littoral, pelagic and profundal habitats, and supported a generally comparable fish fauna dominated by coregonids (S1 Table).

### Focal fish taxa

Previous work in these lakes has firmly established the existence of polymorphic whitefish populations based on ecomorphology and genetic markers, with two to four divergent morphs described within each system [12,34–36]. Morphs are delineated according to gill raker (GR) number and body size, with each morph typically having distinct patterns of resource and habitat use [7,34,37,39]. Densely-rakered (DR) whitefish typically feed on zooplankton in the pelagic zone; large densely-rakered (LDR) whitefish feed on zooplankton and terrestrial insects, mostly in the interface between pelagic and littoral habitats; large sparsely-rakered (LSR) whitefish feed mainly on benthic macroinvertebrates in littoral habitats; and small sparsely-rakered (SSR) whitefish feed mostly on benthic macroinvertebrates in the profundal zone [12,36,39]. The closely related vendace is an introduced pelagic zooplanktivore species and was present in half of the study lakes, providing a model out-group against which to compare the extent of the intraspecific variability across the whitefish morphs [12,37,38].

Brown trout (*Salmo trutta*) and northern pike (*Esox lucius*)—hereafter trout and pike—are among the most common and abundant piscivore species in the study region and show distinct pattern of resource and habitat use [12]. In these lakes, trout and pike shift to piscivory at a total length of circa 27 cm and 20 cm, respectively [40]. Trout is generally more dependent on pelagic than littoral habitats and associated prey species, whereas pike is more common in near-shore areas, though it generally feeds opportunistically on all encountered prey fish [12,13,40,41]. As such, these two species represent two contrasting end-points of trophic pathways via which energy and matter reach the top of the lake food webs.

### Sample collection and preparation

Sampling of fish and invertebrate prey was carried out during late August—early September in 2009, 2010 and 2014. In the Finnish lakes, fish were caught using gill net series comprising of eight 1.8 m high and 30 m long nets with mesh sizes (knot-to-knot) of 12, 15, 20, 25, 30, 35, 45 and 60 mm supplemented with 30 m long and 1.5 m high Nordic multimesh gillnet with 12 mesh sizes from 5–55 mm. In the Norwegian lakes, fish were captured in littoral and profundal habitats with 40 m long and 1.5 m high gill nets with mesh sizes 10, 12.5, 15, 18.5, 22, 26, 35 and 45 mm, whereas the pelagic habitat was sampled with 50 m long and 6 m high gill nets with 6, 8, 10, 12.5, 15, 18.5, 22, 26, 35 and 45 mm (knot-to-knot) mesh sizes. Gill nets were set overnight in pelagic (0–6 m from the surface), littoral (<5 m depth) and profundal (> 8 m depth) habitats. As pike were difficult to capture in some locations using gill nets alone, samples were supplemented with individuals caught via rod and line. We selected recently entangled fish from gill nets, focusing on adult individuals encompassing the typical size range of each morph and species (Table 1). At each site, zooplankton and benthic macroinvertebrates were sampled concurrently with fish to establish habitat-specific isotopic baselines for secondary production. Across all lakes, standardised invertebrate taxa were selected that were highly abundant and considered to be the best representative integrators of primary production in each habitat type: bulk zooplankton including both cladocerans (sum of *Bosmina* sp., *Daphnia* sp. and *Holopedium* sp.; 43% of sample) and copepods (sum of Calanoida and Cyclopoida; 57%) were sampled with semi-vertical (0–15 m) tows using a 250-μm mesh plankton net in the pelagic; chironomidae larvae were sampled from profundal habitats (10–30 m depth) using an Ekman grab (sampling area: 272 cm^2^); *Lymnaea sp*. snails were collected by hand from littoral areas (<1 m), and de-shelled prior to storage. All invertebrate samples were subsequently frozen at -20 °C and freeze-dried at -50°C for 48 hours.

**Table 1:** Sample sizes, total length, gill raker number (GR) and diet of selected fish taxa in lakes of the Paatsjoki watercourse (DR = densely-rakered whitefish, LDR = large densely-rakered whitefish, LSR = large sparsely-rakered whitefish, SSR = small sparsely-rakered whitefish). Diet is divided into littoral, pelagic and profundal groups, with the main prey group for each species and morph highlighted in bold (these values were used as diet priors in subsequent stable isotopes mixing models).

	Brown trout	Pike	DR	LDR	LSR	SSR	Vendace
Sample size	15	18	15	15	18	12	9
Length (cm±1SD)	44.6±7.9	53.5±8.9	14.8±1.8	31.4±2.8	36.1±4.2	24.0±2.4	14.2±1.4
GR (±1SD)			36.5±3.1	34.1±2.7	22.6±2.3	18.1±1.7	43.3±2.3
**Pelagic prey**							
Calanoida	0	0	5.7	3.1	0	0	12.3
*Bosmina* sp.	0	0	38.5	6.6	0	0	61.3
*Daphnia* sp.	0	0	23.1	16.2	0	0	25.5
Copepoda	0	0	18.1	14.6	0	0	0
*Holopedium sp*.	0	0	0	0.6	0	0	0
Cyclopoida	0	0	2.3	0	0	0	0.9
Surface insects	0	0	11.3	56.9	0	2.8	0
DR whitefish	36.2	8.7	0	0	0	0	0
Vendace	28	0	0	0	0	0	0
**Littoral prey**							
*Eurycercus* sp.	0	0	0	0	11.7	9.4	0
Trichoptera larvae	4.9	0	1.1	0.7	0.3	0	0
*Asellus aquaticus*	0	0.1	0	0	21.7	0	0
*Valvata* sp.	0	0	0	0	16.4	0	0
LSR whitefish	24.8	40.4	0	0	0	0	0
9-spined stickleback	6.1	6.6	0	0	44	0	0
Burbot	0	16	0	0	0	0	0
Perch	0	8	0	0	0	0	0
**Profundal prey**							
Chironomid larvae	0	0	0	0.7	5.9	40.9	0
*Pisidium* sp.	0	0	0	0	0	38.3	0
Ostracoda	0	0	0	0.4	0	0.9	0
Megacyclops	0	0	0	0	0	7.5	0
Hydracarina	0	0	0	0.1	0	0	0
SSR whitefish	0	20.2	0	0	0	0	0
Pelagic prey (%)	**64.3**	8.7	**98.9**	**98**	0	2.8	**100**
Littoral prey (%)	35.7	**71.1**	1.1	0.7	**94.1**	9.4	0
Profundal prey (%)	0	20.2	0	1.3	5.9	**87.8**	0

Following capture, all fish were immediately removed from gill nets or other sampling gear, euthanised by cerebral concussion, and transported on ice to a field laboratory for further processing. All fish were identified to species/morph and total length (±1 mm accuracy) was measured. For whitefish and vendace, we dissected the first left gill arch and counted the number of gill rakers for whitefish morph and species identification purposes, as this trait is heritable [31,38] (Table 1).

### Sampling strategy

From the wider pool of all captured fish, representative individuals for each focal taxon (n = 3; though not all taxa occurred in all lakes) were selected from each study lake (n = 6) on the basis of typical body size. For the invertebrates, identical taxa from each lake were selected in order to establish common habitat-specific baselines for subsequent FA and stable isotopic analyses, and here samples comprised homogenised bulk samples composed of at least 10 individuals (in order to reduce the influence on intra-taxon variability). As such, the taxa chosen to represent each habitat in each lake were pelagic zooplankton, littoral *Lymnaea* sp. snails and profundal chironomid larvae. Although the sample sizes here were small, they are comparable to those of other studies using FA analysis [25,27,28], and were a necessity given the extensive analytical procedures involved with extraction and identification of FA from animal tissues, and our desire to incorporate data from a number of lakes in order to asses the generality of any patterns we ultimately observed. To increase statistical power, data from all lakes were combined for subsequent analyses, under the assumption that each taxon exploited similar niches in all systems, a phenomenon identified consistently in earlier studies of these lakes [7,37,40]. All lakes were also broadly similar across a range of physicochemical and ecological variables, and exist in close geographical proximity, suggesting that comparable patterns of resource use are plausible to expect (S1 Table; S1 Fig). Indeed, this assumption was supported by comparable whitefish diet data across the surveyed lakes (S3 Table), and from clustering of individuals belonging to each taxon and their invertebrate prey in isotopic niche space (S2 and S3 Figs), despite the potential for large inter-lake differences in stable isotope values in general.

### Stomach content analyses

The stomach of each fish was dissected and its content determined using a points method; stomach fullness of each sampled individual was estimated visually on a scale from 0 (empty) to 10 (fully extended), with the relative contribution of each prey taxon subjectively evaluated [42]. All prey items were identified to the lowest feasible taxonomic level (see Table 1), based on the extent of digestion. Prey were further classified into pelagic, littoral and profundal categories based on predominant patterns of habitat utilisation and occurrence within the lakes [43,44]. For each individual, a sample of white muscle tissue for stable isotope and FA analyses was taken laterally, posterior to the dorsal fin, and frozen at -20 °C and subsequently freeze-dried at -50°C for 48 hours.

### Stable isotope analysis

Freeze-dried samples were ground, and 0.5–1 mg subsamples were encapsulated in tin cups. All samples were analysed for carbon and nitrogen stable isotope ratios using an elemental analyser coupled to a continuous-flow stable isotope-ratio mass spectrometer. Across all runs, the standard deviation of laboratory working standards was < 0.2‰ for δ^13^C and ~ 0.2 ‰ for δ^15^N. Stable isotope values were expressed in delta (δ) notation, where quantities of each isotope are expressed as parts-per-thousand (‰) deviation from international standards (Vienna Pee Dee Belemnite for carbon and atmospheric air for nitrogen). As we planned to investigate correlations between FA contents and isotopic ratios, we did not correct data for total lipid content, and C:N ratios (a proxy for total lipid content) were uniformly low in all fish (mean C:N = 3.26; max C:N = 3.87).

### Fatty acid analysis

Lipids were extracted from freeze-dried dorsal muscle tissue samples (ca. 20 mg dry weight) using chloroform:methanol (2:1) as described elsewhere [45]. Total lipid extracts were esterified to obtain fatty acid methyl esters (FAME) using toluene (1 mL) and H_2_SO_4_-methanol (2 mL; 1% v/v). FAME were analysed using a gas chromatograph (TRACE GC THERMO) equipped with flame-ionization detection, a temperature-programmable injector, and an autosampler. A Supelco SP-2560 column (100 m, 25 mm i.d., 0.2 μm film thickness) was used for FAME separation. FA mass ratios were calculated using calibration curves based on known standard concentrations expressed as μg FAME • mg dry weight sample^-1^, with these values then subsequently standardised to relative proportional values (%) for use in further analyses.

### Data analysis

All statistical analyses were performed in R version 3.2.0 [46]. The Stable Isotope Analysis in R (*SIAR*; version 4.2 [47]) mixing model was used to assess the relative proportional contribution of littoral, pelagic and profundal resources to each fish morph/species, based on δ^13^C and δ^15^N. Mean (± 1SD) δ ^13^C and δ ^15^N values of invertebrates in each habitat were used as baselines. Trophic enrichment factors (TEFs) of 0.4 ± 1.3 ‰ for δ^13^C and 3.4 ± 1 ‰ for δ^15^N were assumed for invertebrate-feeding whitefish morphs and vendace, whilst these values (and their associated error) were doubled for piscivorous trout and pike (i.e. 0.8 ± 2.6 ‰ for δ^13^C and 6.8 ± 2 ‰ for δ^15^N), as these species were assumed to be two trophic levels higher than invertebrate primary consumers [48]. As diet data were available for all sampled fish, prior probability distributions of pelagic, littoral and profundal contributions to the diet of each species (Table 1) were constructed using the *siarelicit* function in the *SIAR* package. These priors were used to inform subsequent model fits. All models were then run using the *siarmcmcdirichletv4* function, based on 500,000 iterations, with the first 50,000 discarded [47].

All FA data were analysed as relative proportional values (%) to standardise for different lipid content across taxa and reduce statistical noise. In order to assess whether whitefish morph FA profiles could be readily distinguished from one another (and from their congener, vendace), we used Linear Discriminant Analysis (hereafter “LDA”) on relative proportional values (%) (S2 Table). This supervised classification method uses an *a priori* grouping variable and attempts to project data points into multiple linear dimensions that maximise the dissimilarity between the given groups. However, as our full suite of FA data was highly collinear (S4 Fig) and the dataset included more independent variables than cases (likely leading to over-fitting in this type of model), several pre-processing steps were required prior to carrying out further LDA analyses.

Firstly, as several later steps used iterative subsets of the full dataset that could not run if all values were zeroes, we identified and removed FA variables with <10 non-zero values. Next, all remaining FA data were scaled, centred, and box-cox transformed (if necessary), using the *preProcess* function in the R package *Caret* [49] to improve fitting in subsequent variable selection and LDA steps. Variable selection was then performed on this transformed dataset by fitting a Learning Vector Quantization (LVQ) model using the *varImp* function in the *Caret* package. This generated a ranked list of the top 20 most important variables distinguishing the coregonid groups in the dataset.

As the prior step did not account for collinearity within the dataset, we subsequently performed ascendant hierarchical clustering using the *hclustvar* function in the R package *ClustOfVar* [50] to assess the correlation structure of the data. This method was used to sort the full suite of FA variables into a series of collinear groupings from which a series of indicator variables could then be chosen (see S5 Fig). An optimal number of clusters was then determined *post-hoc* based on 500 iterations using the *stability* function within *ClustOfVar*. Information on cluster membership and variable importance was then combined to provide a set of less strongly correlated “indicator” variables that were used in all subsequent analyses. Here, we chose the most “important” variable from each collinear cluster based on the list of the top 20 list generated by Caret’s *varImp* function (see S4 Table for full group membership and importance ranking information). Clusters where none of the variables appeared in the top 20 list were deemed likely unimportant for distinguishing groups and therefore excluded. This final set of less correlated indicator variables was then used to assess group-wise differences in FA composition among our coregonid taxa.

Once this final set of selected variables was constructed, LDA was carried out using the *lda* function in the package *MASS* [51], using a leave-one-out cross-validation step (assuming proportional priors) to allow for *post hoc* evaluation of predictive performance. Data were subsequently plotted in LD1 vs. LD2 space, with hull areas calculated for each individual whitefish morph, overall for whitefish, and for vendace to assess the overall LDA space occupied by each taxon. The significance of differences in group centroids in this LDA space were determined using PERMANOVA (using the *adonis* function in the R package *vegan* [52], based on 4999 permutations), with *post-hoc* pairwise tests used to test group by group differences. Differential dispersion (i.e. contrasting mean distances to group centroids) was evaluated using the *betadisper* function in *vegan*. To explore which FAs ultimately contributed most to this final LDA, we used a stepwise LDA procedure (using the *train* function in *Caret* with a *stepLDA* method argument) to eliminate all but the most vital variables distinguishing groups. Group-by-group ANOVAs and Tukey’s HSD tests were then used to assess differences in mean levels of these key FAs at each trophic level.

This final set of most important FAs identified by stepwise LDA were then related to isotopic gradients to explore the extent to which these correlated with resource use gradients. All key FAs were modelled against δ^13^C and δ^15^N values in coregonids, with model selection subsequently performed using the *dredge* function within the R package *MuMIn* [53]. This function constructs all possible candidate sub-models nested within the global model, identifies the most plausible subset of models for each data set, and then ranks them according to corrected Akaike Information Criterion (AICc) values and AIC likelihood weights (AICcw). The model with the lowest AICc value was then used to draw statistical conclusions. Finally, in order to assess trends across trophic levels, mean pelagic reliance generated from *SIAR* models was compared to log-transformed levels of the most important FA across all fish taxa using the same model selection approach.

## Results

**Fig 1:**
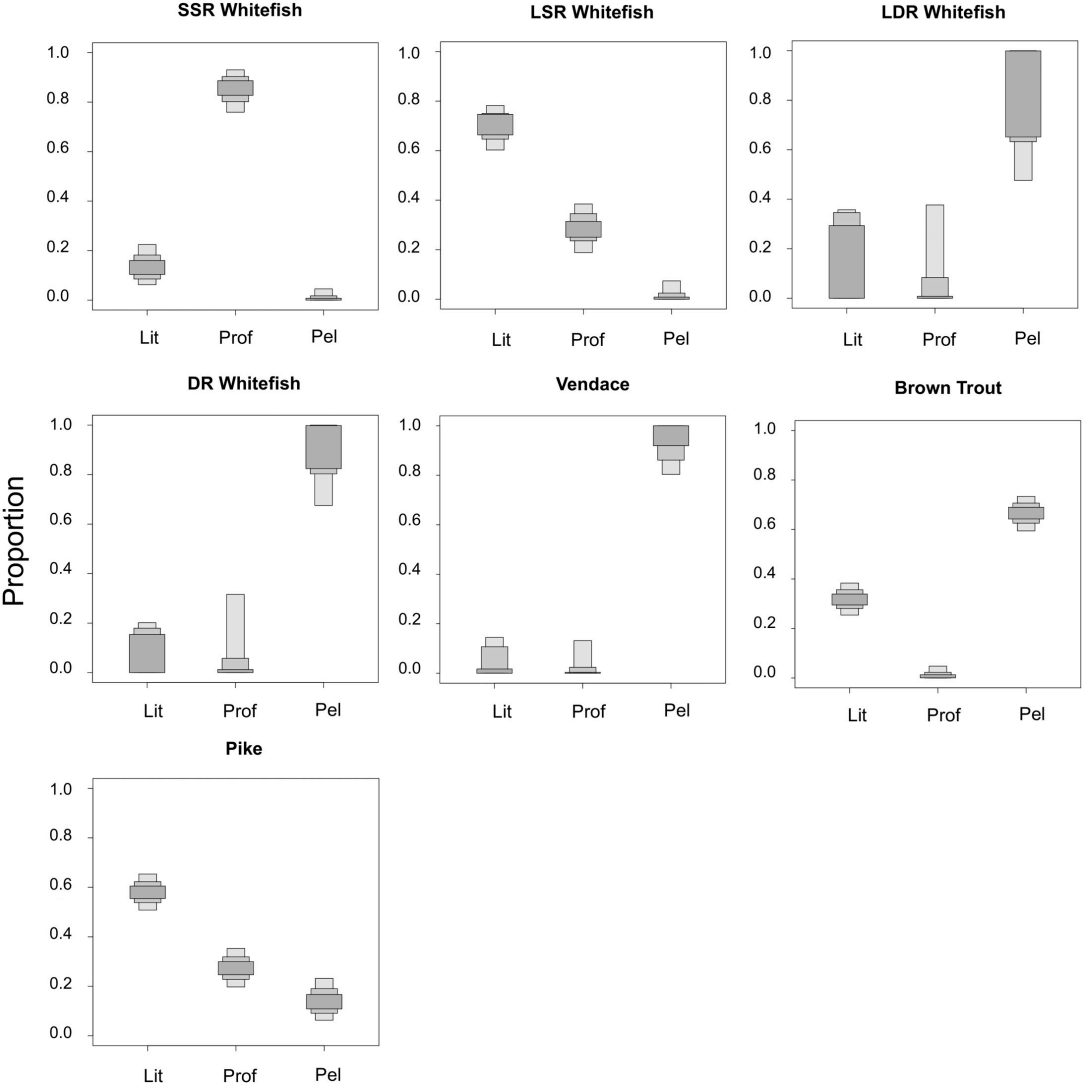
Credibility plots of proportional use of littoral (Lit), profundal (Prof) and pelagic (Pel) resources by seven fish species in lakes of the Paatsjoki watercourse, based on estimates by the Stable Isotope Analysis in R (SIAR) package. Bars represent 95%, 75% and 50% credibility intervals around mean estimates derived from 500,000 iterations, with the first 50,000 discarded.

Both stomach content data and the estimates provided by the SIAR mixing models revealed consistent patterns of distinct littoral, pelagic and profundal resources use across all fish species in general, and among the whitefish morphs specifically (Table 1; Fig 1). LSR whitefish was predominantly reliant on littoral prey taxa, including *Asellus aquaticus*, *Valvata* sp. snails and nine-spined stickleback (diet proportion/mean SIAR littoral estimate: 94.1% / 69.2%), whereas DR whitefish, LDR whitefish and vendace were more dependent on pelagic zooplankton and surface insects (diet/mean SIAR pelagic estimates: DR—98.9% / 87.0%, LDR -98.0% / 75.7%, vendace—100% / 91.1%; Fig 1, Table 1). SSR whitefish was the only morph using mainly profundal resources—such as chironomid larvae and *Pisidium* clams—as a major food source (diet/mean SIAR profundal estimate: 87.8% / 84.8%). Patterns of resource use differed between the two piscivorous species, with trout predominantly reliant on pelagic prey fish, i.e. vendace and DR whitefish (diet/mean SIAR pelagic estimate: 64.3% / 66.5%), whilst pike consumed fish from all three habitats, though with a pronounced dependence on littoral LSR whitefish, perch and burbot (diet/mean SIAR littoral estimate: 71.1% / 58.1%, Fig 1, Table 1).

**Fig 2:**
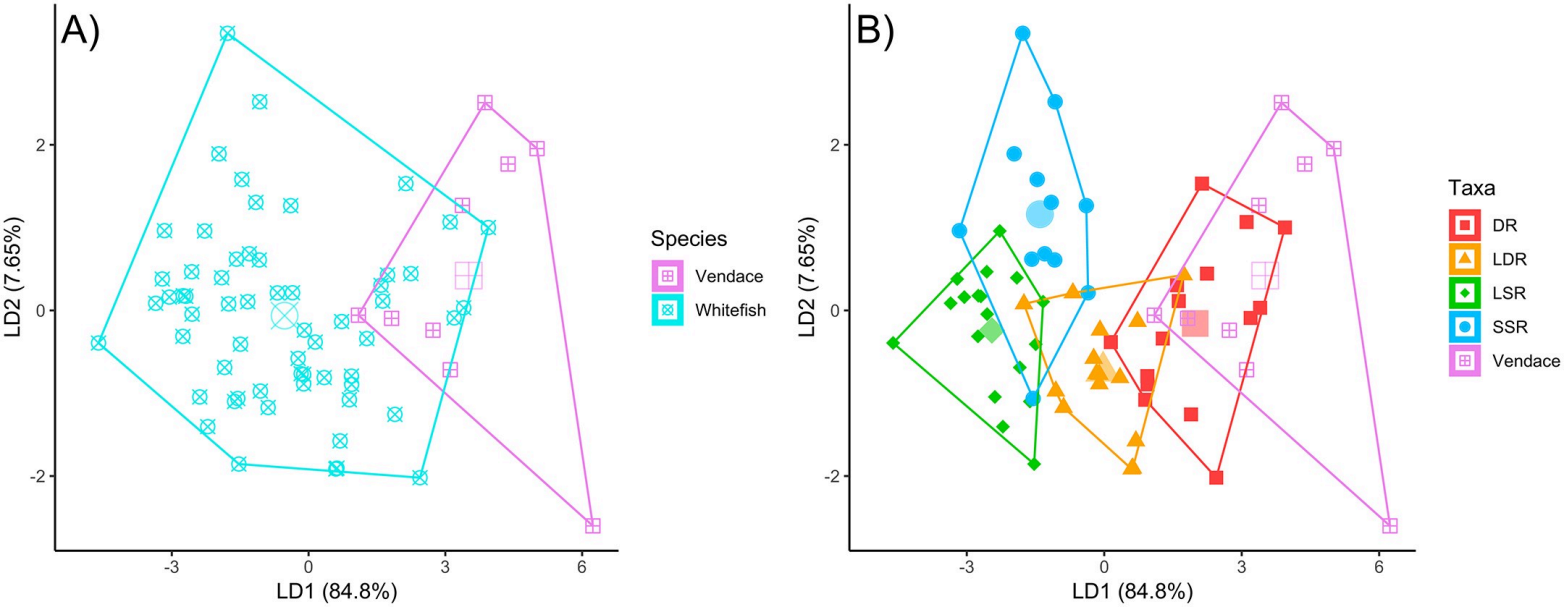
Plots of the first two LDA axes for all coregonid taxa. Hulls indicate grouping; left-hand plot (A) shows species-level differences between whitefish and vendace; right-hand plot (B) show differences among whitefish morphs. Larger translucent points represent group centroids. Percentage of total variance explained by each axis is included in brackets. DR = densely rakered whitefish, LDR = large densely rakered whitefish, LSR = large sparsely rakered whitefish, SSR = small sparsely rakered whitefish.

Our combined learning vector quantification feature selection and ascendant hierarchical clustering approach reduced the full 46 FA dataset substantially; the former generated a ranked list of the 20 most important variables separating the coregonid taxa (S4 Table), whilst the latter suggested that there were 11 main clusters present within the dataset (S6 Fig). Together, these two methods resulted in a reduced dataset containing seven major FAs (S4 Table), which were substantially less collinear than the full suite of variables (see S7 Fig) and improved subsequent model fitting. LDA performed on this reduced set of less collinear variables was able to discern groups fairly readily in low dimensional space (Fig 2), with the first two LD axes explaining > 92% of the total variance (LD1: 84.8%; LD2: 7.65%). As these two axes together explained a large majority of the overall variance, they were used in all subsequent analyses. These axes described a pronounced gradient, with the lowest LD1 values belonging to benthic coregonid taxa and increasing values associated with increasingly pelagic ones (Fig 2)–mirroring patterns seen in diet and SIA analyses (Fig 1). Trends along LD2 were less pronounced, but seemed to indicate a vertical depth gradient, where lower values were related to shallow littoral or near surface habitat (LSR and LDR) and higher values increasing depth (SSR and vendace). Leave-one out cross validation using information from the full suite of LD axes revealed that classification success was reasonable given our relatively low samples sizes, with 81% percent of individuals successfully assigned correct groupings based on their FA profile (Table 2). Here, LSR whitefish appeared the most distinct group, and were never confused with other taxa. Conversely, a third of vendace were incorrectly classified as DR whitefish, and one DR whitefish specimen was incorrectly identified as vendace. There also appeared to be some overlap between LDR and SSR whitefish.

**Table 2:** Classification table based on a leave-one-out cross validation linear discriminant analysis of select FA in coregonid taxa. Model assumed proportional priors and estimation was based on n = 69 cases. Overall accuracy: 81.16%.

	**Predicted**				
**Observed**	DR	LDR	LSR	SSR	Vendace
DR	**13**	1	0	0	1
LDR	1	**12**	0	2	0
LSR	0	0	**18**	0	0
SSR	0	3	1	**8**	0
Vendace	3	1	0	0	**5**

Coregonid taxa occupied varying amounts of LDA space, with vendace occupying the most (12.39 squared LD units), whilst individual whitefish morphs tended to vary less (DR: 7.14; LDR: 4.58; LSR: 4.79; SSR: 6.96). However, at the overall species-level, whitefish occupied almost 2.5x the LD space that vendace did (28.54 vs. 12.39 LD units). The overall PERMANOVA revealed that several group centroids were significantly different within LD1 vs. LD2 space (F_4,64_ = 41.18; p < 0.001), with pairwise tests revealing that all groups were significantly different from each other (p < 0.05 in all cases; S5 Table). Betadisper indicated that there was significant unequal dispersion among groups (F_4,64_ = 3.67; p < 0.009), with vendace being more widely dispersed from their group centroid than both LSR (p = 0.010) and LDR (p = 0.009) whitefish. However, when considered at an overall species-level, vendace and whitefish did not show differential dispersion (F_1,67_ = 0.02; p = 0.881).

**Fig 3:**
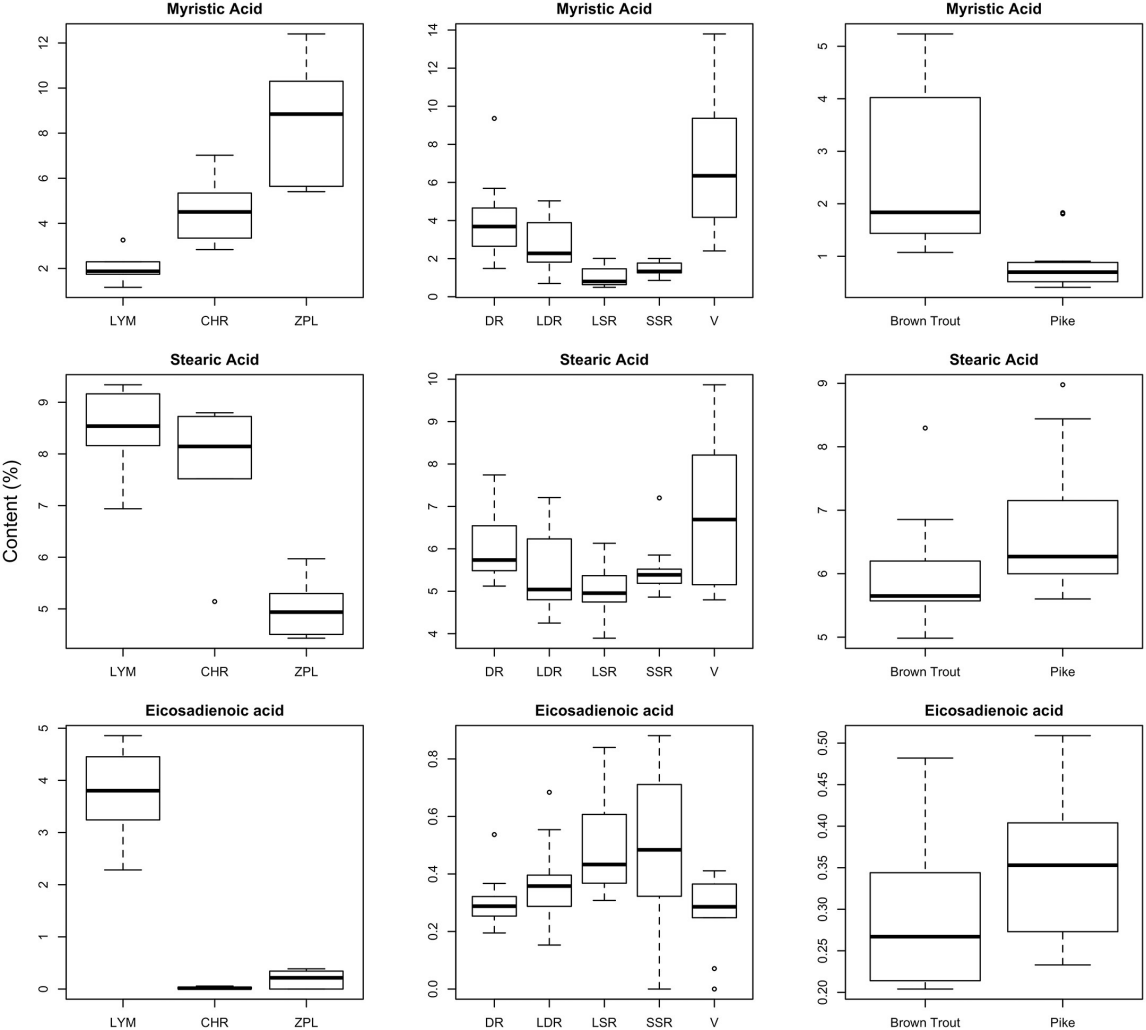
Boxplots of percentage concentrations of the three major fatty acids segregrating coregonid taxa across all three sampled trophic levels. LYM = *Lymnaea* sp., CHR = profundal chironomids, ZPL = zooplankton, DR = densely-rakered whitefish, LDR = large densely-rakered whitefish, LSR = large sparsely-rakered whitefish, SSR = small sparsely-rakered whitefish, V = vendace.

The StepLDA model revealed that among our seven most important FAs, some were even more useful in distinguishing coregonid groups than others, with the final model here containing only three FAs: myristic acid (14:0), stearic acid (18:0) and eicosadienoic acid (20:2n-6). As such, variation in these three FAs was explored across coregonid groups and their wider food webs. Levels of myristic acid were generally higher in pelagic coregonids than in littoral ones (Fig 3), and levels varied significantly across groups (F_4,64_ = 19.67; p < 0.001; S6 Table); vendace had significantly higher levels than all whitefish morphs (p < 0.01 in all cases), but intraspecific differences were also apparent within the whitefish morphs, with DR whitefish having higher levels than LSR and SSR whitefish (p < 0.01 in both cases), while LDR whitefish were intermediate. ANOVA (F_4,64_ = 6.80; p < 0.001) revealed that vendace had higher levels of stearic acid than LDR, SSR and LSR (p < 0.01 in all cases), though had levels indistinguishable from DR whitefish (p = 0.225). DR had higher levels than LSR (p < 0.05). Eicosadienoic acid levels also varied across coregonid groups (F_4,64_ = 6.31; p < 0.001), with levels highest in benthic LSR and SSR whitefish and significantly lower in vendace and DR whitefish (p< 0.05 in all cases). LDR were intermediate (p > 0.05 compared to all other taxa).

The differences in the three key FAs across whitefish morphs utilising different lake habitats was also largely mirrored in their invertebrate prey (Fig 3; S6 Table). For instance, myristic acid levels were significantly higher in pelagic zooplankton than they were in profundal chironomids (p < 0.01) or littoral *Lymnaea* (p < 0.001), while levels of stearic acid showed the opposite pattern, being lowest in zooplankton compared to the other two invertebrate groups (p < 0.001 in both cases). Levels of eicosadienoic acid were significantly higher in *Lymnaea* than in the other two invertebrate groups (p < 0.001 in both cases). Relationships in the two piscivore species also seemed to indicate that these patterns were consistent throughout the food web (Fig 3), with myristic acid levels higher in pelagic brown trout than littoral pike (p<0.001), while the opposite trend was apparent for stearic acid (p = 0.031) and differences in eicosadienoic acid were marginal (p = 0.051).

**Fig 4:**
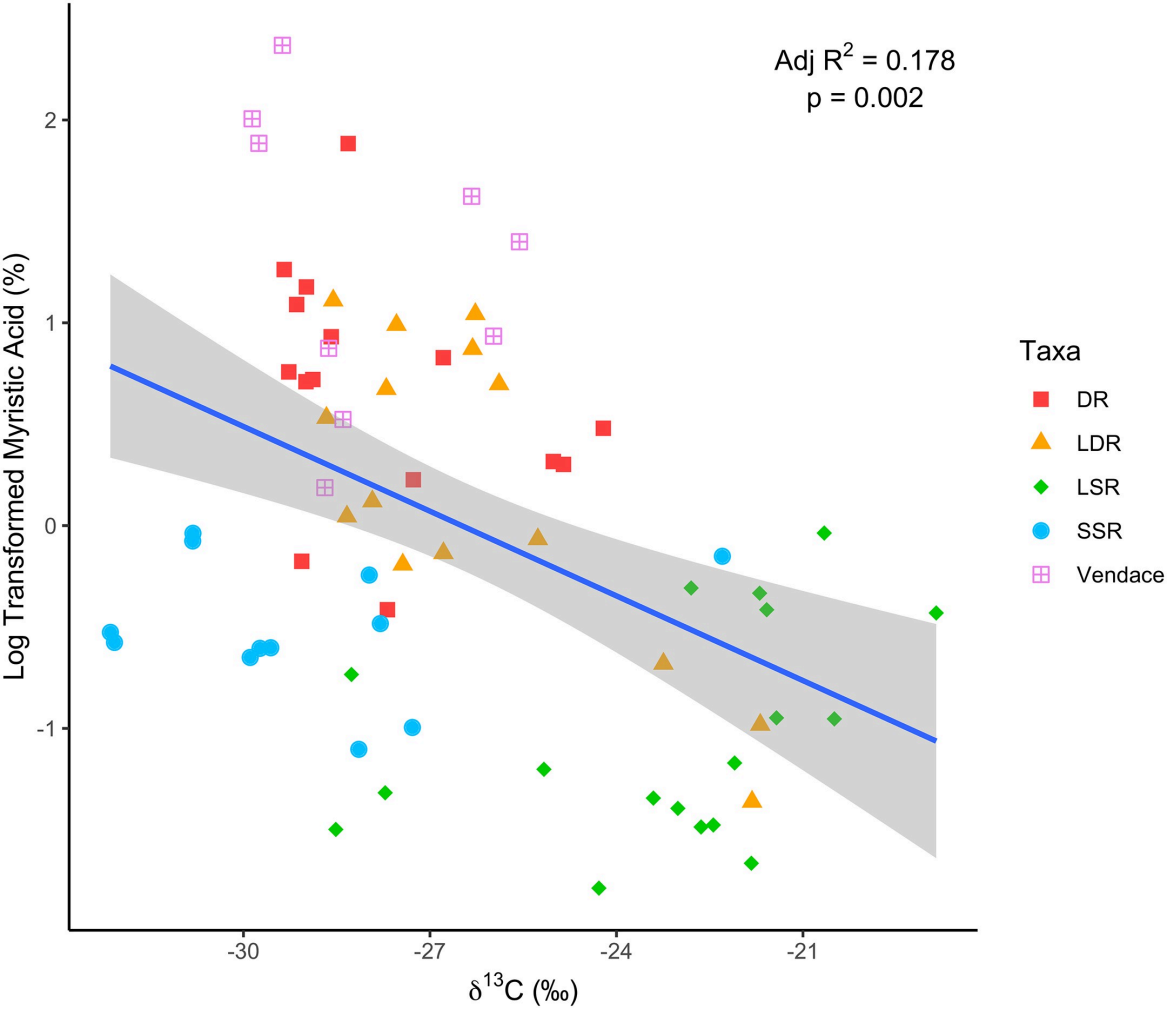
Relationship between log-transformed myristic acid concentration (%) and δ^13^C (‰) in coregonid taxa. DR = densely-rakered whitefish, LDR = large densely-rakered whitefish, LSR = large sparsely-rakered whitefish, SSR = small sparsely-rakered whitefish.

The dredge function used to assess relationship between key FAs and δ^13^C and δ^15^N revealed relationships between these major tracers. The overall δ^13^C global model containing all three variables was significant (F_3,65_ = 6.64; p < 0.001). The best model (based on AICc scores) resulting from dredging the δ^13^C model included myristic acid (14:0) and stearic acid (18:0), and explained 20.4% of the overall variance (F_2,66_ = 9.75; p < 0.001). However, in this final model, only myristic acid (14:0) showed a significant relationship with δ^13^C (Fig 4; F_1,66_ = 10.36; p < 0.001), while stearic acid (18:0) was only marginal (F_1,66_ = 3.21; p = 0.078). None of the FA correlated with δ^15^N and the global model here was not significant (F_3,65_ = 0.77; p = 0.516).

**Fig 5:**
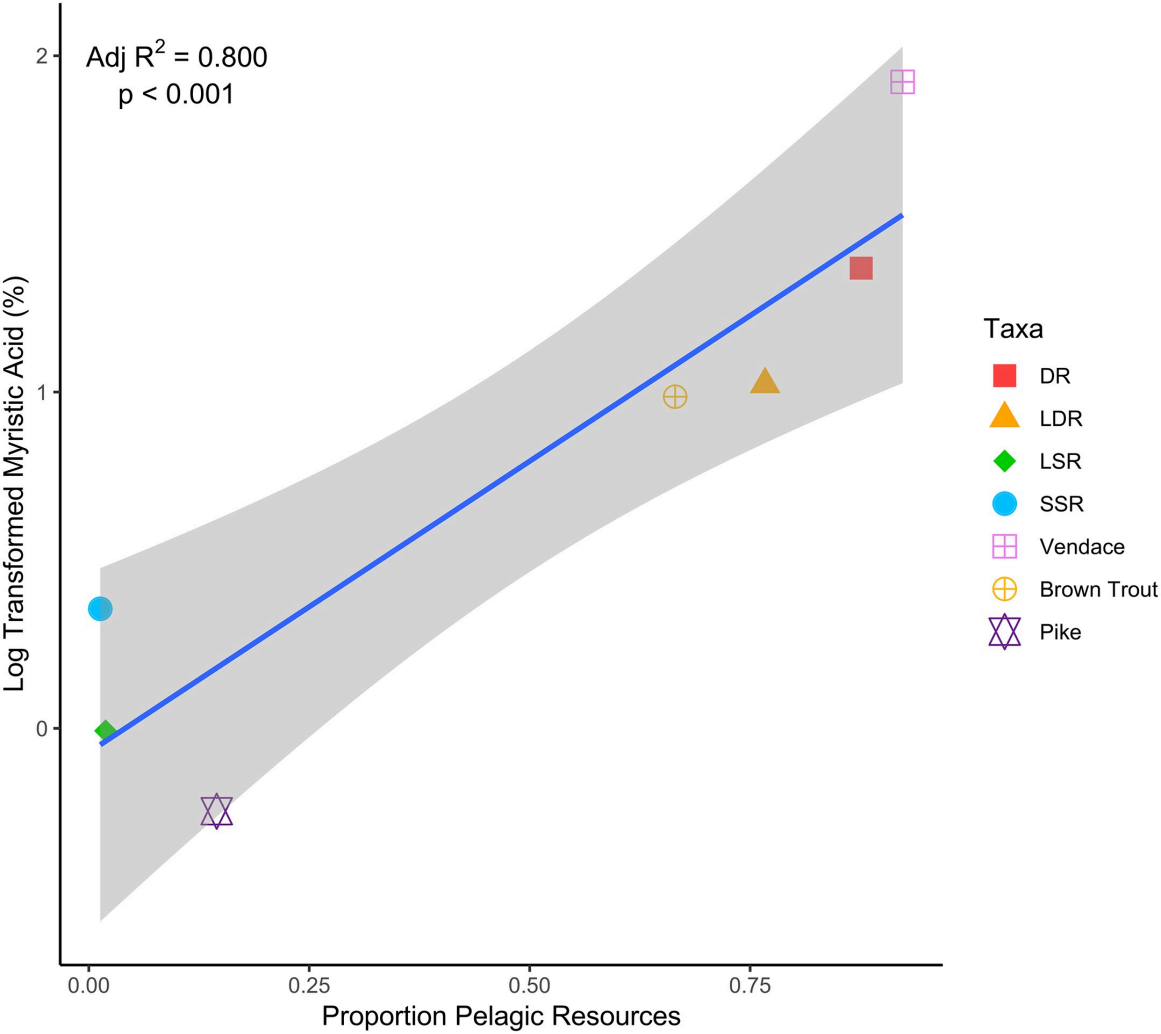
Relationship between log-transformed myristic acid concentration (%) and mean pelagic reliance (estimated from SIAR model based on δ^13^C, δ^15^N and diet data) across all fish taxa. DR = densely-rakered whitefish, LDR = large densely-rakered whitefish, LSR = large sparsely-rakered whitefish, SSR = small sparsely-rakered whitefish.

Relationships were also strongly evident at the fish community level when population mean pelagic reliance estimates generated by SIAR were considered. An overall global model containing all three variables was significant (F_3,3_ = 21.41; p = 0.016). The best model (based on AICc scores) resulting from dredging contained only myristic acid, though the relationship here was strong (Fig 5; F_1,5_ = 25.06; p = 0.004).

## Discussion

In the present study we combined data from three distinct trophic markers, enabling us to reveal clear evidence for resource polymorphism across the whitefish populations of our Fennoscandian study lakes, confirming patterns seen in prior ecomorphological and genetic analyses [7,34,35,37]. Four distinct morphs exploited the three principal lake habitats across depth and littoral-pelagic gradients, with resultant differences apparent in their trophic ecology and tissue composition, supporting our initial hypotheses. Moreover, we show that FA analysis can delineate polymorphic trophic groups with even greater resolution than traditional diet and stable isotope methods, (as they provided the clearest segregation between trophically-similar DR and LDR whitefish,) and also has the capacity to reveal distinct patterns of dietary energy transfer throughout complex lake food webs. When information from all three analyses utilised here was combined, trophic niche segregation among pelagic, littoral and profundal fish was highly consistent, irrespective of taxonomic identity. δ^13^C values correlated with resource use along the pelagic-benthic resource axis, whilst δ^15^N values increased with trophic level throughout the lake food webs. Two FA—myristic acid (14:0) and eicosadienoic acid (20:2n-6)–documented a pelagic-benthic habitat use gradient throughout the food web, as evidenced by tissue concentrations in invertebrates, coregonids and their predators, whilst evidence from a third, stearic acid (18:0) was somewhat less clear. Taken together, these findings highlight the utility of a combined diet, stable isotope and FA approach to infer patterns of trophic niche segregation with high resolution, even within populations of the same species adapting to differential resource use.

European whitefish is noteworthy for its ecological diversity [31,32,54], and the present study confirmed distinct dietary segregation among the four whitefish morphs, a pattern observed directly via stomach contents. The most ubiquitous whitefish morph in the region, LSR whitefish, was a benthivore, consumed a wide range of littoral prey items, whereas the other benthivore, SSR whitefish, relied mainly on profundal prey items such as chironomid larvae and *Pisidium* clams. Notably, the two pelagic morphs showed partial niche segregation, with the small-sized DR whitefish using smaller cladoceran prey than large-bodied LDR whitefish, which predominantly predated surface insects and copepods. Vendace had the largest dietary overlap with its congener DR whitefish, with both reliant on a similar cladoceran-dominated diet. These two planktivores were heavily consumed by pelagic trout, whereas littoral pike exploited LSR whitefish as their major prey resource. In addition to largely supporting the patterns revealed from direct observation of stomach contents, dietary FA and stable isotope data revealed two relatively distinct food web compartments within the lake: one derived from littoral primary production and supporting benthic invertebrates, LSR whitefish and finally pike, and a second pelagic phytoplankton-based pathway supporting zooplankton, planktivores, and ultimately trout. The profundal food web appeared to rely mostly on pelagic-derived settling matter (based on δ^13^C ratios), as evidenced by enriched δ^15^N ratios in chironomids and especially SSR whitefish—the latter of having δ^15^N values equivalent to piscivorous trout and pike. Energy transport in this profundal compartment usually ends with SSR whitefish, as there is little predation in these dark, cold and unproductive deep-water environments, as manifested in the morph’s life history strategy, with distinct adaptations including slow metabolism, high longevity and late maturation compared to the other morphs [36,40,43,44].

When FA data were used in conjunction with dietary and isotopic analyses, we found even stronger evidence of trophic niche segregation among whitefish morphs and vendace than could be obtained from traditional analyses alone, particularly between taxa where trophic niche overlap was high. Here, a major portion of overall variance (>92%) was explained by the two first to LD axes: LD1 (84.8% of variance) provided overall benthic-pelagic axis ranging from LSR whitefish to vendace, whilst LD2 (7.7%) appeared to provide some evidence of diversification related to the lakes’ vertical depth gradients, where SSR whitefish and vendace use deeper feeding habitats than other morphs. These observations accurately concur with previous trophic ecological studies of these morphs and species [7,12,13,35,39], but extend our knowledge on their finer trophic niche segregation, especially in pelagic habitats. Compared to stable isotope ratios, mercury content and dietary evidence of the same morphs and species [7,12,13,35,39], we found that LDR whitefish in particular could be identified more effectively based on their FA profile (although they were still occasionally confused with other taxa). This morph feeds on both pelagic zooplankton and surface insects (terrestrial and aquatic), actively cruising close to the surface both in pelagic and littoral areas [13,36,39], which correlates well with its FA composition relative to LSR and DR whitefish. While our sample size is somewhat limited and distributed among several lakes, the overall consistency in patterns of FA composition in whitefish morphs and vendace suggest common dietary and physiological processes are likely operating across all of our study systems. In general, our FA results support a gradient of pelagic and benthic feeding taxa [13,39], where LSR whitefish represent one end and vendace the other. As resource polymorphism is especially common across pelagic and littoral resource gradients [55], our results probably warrant further study of FA composition in other comparable polymorphic systems. We suggest future studies might compare stable isotope and FA niche sizes of whole fish communities by using larger sample sizes (optimally 30+ individuals per taxon per lake). This would provide interesting opportunities to assess the efficacy of the combination of these two methods to explore biotic links among the species and energy flows throughout pelagic and benthic compartments more explicitly. At a more detailed level, the compound specific stable isotopes of identified key FAs may give further insights to trophic niche segregation among morphs and species [56].

Specific FA were identified as particularly useful for understanding trophic interactions in our study lakes, with myristic acid (14:0), stearic acid (18:0) and eicosadienoic acid (20:2n-6) proving to be the major compounds effectively segregating pelagic—and benthic—reliant taxa. These results highlight the potential of saturated FA—particularly 14:0 and 18:0 –as trophic biomarkers. In fish, all of these FA had low mass ratios compared to DHA, which is often the dominant FA in fish tissues [10,45]. Due to the high retention of DHA across all studied fish species, this FA was of little value as a biomarker and was not one of the key FA readily differentiating pelagic and benthic species based on our analyses. In contrast, less abundant FA, such as myristic and stearic acid—which typically have low bioconversion rates in fish—proved to be characteristic of differential littoral-pelagic resource use [11,26,28,30]. Myristic acid was high in zooplankton, planktivores (vendace and DR whitefish) and brown trout compared to benthic macroinvertebrates, benthivores (LSR and SSR) and pike. Although all phytoplankton taxa produce myristic acid, high levels are typical in diatoms, which often dominate the pelagic algal communities of oligotrophic lakes [21,57,58] and support zooplankton production [20], and were likely highly abundant within the phytoplankton fauna of the study systems. The value of myristic acid as a biomarker seems to be high in a wide variety of aquatic environments; this compound has previously been found to identify pelagic feeding by herring gulls (*Larus argentatus*) in both marine [11] and lake ecosystems [26], and proven important in segregating morphs of *Percichthys trucha* in South America [25] and *Salvelinus namaycush* in the North American great lakes [29], whilst also proving important to differentiate pelagic coregonids from other fish in Lake Baikal [28]. This suggests the likely generality of myristic acid as a key biomarker in identification of divergence along pelagic-benthic resource axes in oligotrophic lakes, and may be particularly useful when combined with mutually supportive evidence from stable isotope data.

Aside from myristic acid, the other major FA differentiating pelagic and benthic resource use was eicosadienoic acid. Content of eicosadienoic acid was high in littoral *Lymnaea* snails, in whitefish morphs predating benthic macroinvertebrates (LSR and SSR whitefish) and in piscivorous pike. This may indicate a littoral flow of energy and biomolecules originating from periphyton consumed by snails and other littoral consumers. However, eicosadienoic acid content of profundal SSR whitefish is unlikely to be linked to consumption of littoral benthic prey, as this morph is a deep-water specialist. Stearic acid content was high in *Lymnaea*, profundal chironomids, DR whitefish, vendace and pike. As stearic acid is typically one of the major FA present in both pelagic and benthic primary producers [21,24] it may be a major residual component in the FA of settling organic matter from decaying phytoplankton or terrestrial particulates that are subsequently ingested by both littoral and profundal consumers [59]. Although different fish taxa were delineated by stearic acid content in our study lakes, the pattern from invertebrates to top consumers was not clear. Prior evidence suggests that stearic acid is often produced across the whole pelagic-benthic axis and often abundant in all studied species, and therefore may be of limited use as source-specific biomarker [11,28,60].

Within the study lakes, FA analysis provided detailed insights into both the biochemical composition of consumers across multiple trophic levels, and an increased understanding of the origins of their predominant energy sources. Our sampling was conducted in late summer, close to maximum water temperatures and zooplankton abundance, which typically coincide with the peak growth season and period of highest annual body condition in fish populations [44,61,62]. Content of myristic acid, eicosadienoic acid and stearic acid in pelagic whitefish morphs were within the range documented for other pelagic whitefish in alpine lakes [63,64]. However, DHA mass ratios in these cold, oligotrophic lakes were generally higher than those in more southern lakes. Although our FA results suggest some compounds effectively segregate pelagic and benthic morphs in these subarctic lakes, it is likely that FA profiles may vary temporally, with variation likely being most pronounced following late autumn spawning and the subsequent period of starvation under ice cover [56,62,65–67]. Rates of turnover in FA composition are likely to be less predictable than those in stable isotope ratios (which follow distinct decay curves when consumers switch diets [15]), likely due to variation dependent upon the relative availability of specific FA in the consumer’s environment (wherein rarer compounds are more likely to be retained than metabolized, therefore functioning better as biomarkers), coupled with the effects of differential energetic and bimolecular routing among tissues [56,68]. For instance, the FA composition of highly metabolically active tissues such as gonads and liver are likely to change more rapidly than those of storage tissues such as muscle and perivisceral adipose [62,68]. In sexually mature lake whitefish and vendace, FA investment in eggs is often particularly pronounced, with, for example, certain FA concentrations in reproductive tissues 2–3 times higher than those of muscle tissue [65,66]. In contrast, our sampling of white muscle tissue at the end of the growing season should closely correlate with major dietary-induced differences in FA profiles among our studied taxa, which might have become less pronounced closer to the spawning season [61,65,67,69].

In combination, the methods utilised in this study allowed us to uncover detailed patterns of trophic niche segregation among our coregonid taxa, supporting genetic and morphological evidence that resource polymorphism was indeed present in this system. Together, detailed stomach content prey identity information, stable isotopic information and consumer FA profiles provided for a complementary and integrated view of these subarctic lake food webs, allowing us to delineate niche partitioning at increasingly finer scales; of the three methods, results from FA provided the most detailed segregation of whitefish morphs, particularly in cases where trophic niche use was known to be most similar. Myristic acid derived from pelagic sources was an important trophic biomarker, both in segregating coregonid taxa and in indicating the degree of pelagic-benthic resource use across the wider food web, and may prove similarly effective to δ^13^C in delineating such patterns in lakes. Based on our findings, we argue that data on characteristic FA content in consumer tissues appear to provide more detailed evidence on trophic niche use compared to dietary and isotopic analyses alone, with the higher dimensionality of the datasets generated using this method allowing for a more fine-scale analysis of taxon-by-taxon differences. Inclusion of FA data in analyses of lake trophic ecology may help bridge the gap between dietary and isotopic methods in terms of taxonomic resolution and temporal consistency, and increase the ability to resolve trophic relationships both within polymorphic species specifically, and throughout aquatic food webs more generally. Further studies are required to assess the general utility of FA in delineating such polymorphic groupings in other species, but we suggest our results provide compelling evidence of the utility of such integrated approaches, both for identifying patterns of resource polymorphism in post-glacial fishes, and for studying trophic dynamics in complex freshwater ecosystems more broadly.

## Supporting information

S1 Table Background data on the spatial location, morphometry, physical chemistry and fish fauna of study lakes in the Paatsjoki watercourse. (DOCX)

S2 Table (see attached Excel sheet): Fatty acid concentrations (%) in invertebrates and fish sampled from six lakes in the Paatsjoki watercourse, Fennoscandia. Important fatty acids delineating coregonid taxa and their wider food webs are indicated in bold, whilst final variables selected for use in subsequent analyses are indicated in red.
(XLSX)

S3 Table (see attached Excel sheet): Upper table = Morphological and diet data for each individual fish included in this study; values for diet data represent proportional presence of each prey within the stomach of each individual, based on a point-based method (see: *Sample collection and preparation)*. Lower table = average percentage totals for stomach contents of each study taxon.
(XLSX)

S4 Table (see attached Excel sheet): Relative importance ranking and cluster membership of the 20 most important fatty acids delineating coregonid taxa. Final variables selected for use in subsequent analyses are indicated in red.
(XLSX)

S5 Table (see attached Excel sheet): Results of PERMANOVA tests comparing coregonid taxa in LDA space. Overall tests for species and taxa comparisons are included, along with pairwise tests for whitefish morph and vendace.
(XLSX)

S6 Table (see attached Excel sheet): Results of ANOVA and Tukey’s HSD analyses for differences in concentration of the three main fatty acids identified by a stepwise LDA procedure. (XLSX)

S1 Fig Study area map illustrating northern Fennoscandia (a) and Paatsjoki-Pasvik watercourse with six study lakes (b). (TIFF)

S2 Fig Stable isotope biplots showing individual δ^13^C and δ^15^N for each sampled fish taxon. Symbols indicate individual lakes: Lake Inari = dark green circles; Lake Muddus = orange circles; Lake Paadar = purple circles; Lake Ukko = pink circles; Lake Vaggatem = light green circles; Lake Vastus = yellow circles. NB: Not all taxa occur in all lakes.
(TIFF)

S3 Fig Stable isotope biplots showing individual δ^13^C and δ^15^N for invertebrate baselines. Symbols indicate individual lakes: Lake Inari = dark green circles; Lake Muddus = orange circles; Lake Paadar = purple circles; Lake Ukko = pink circles; Lake Vaggatem = light green circles; Lake Vastus = yellow circles. NB: Not all taxa occur in all lakes.
(TIFF)

S4 Fig Correlation plot for the full suite of 46 fatty acids analysed as part of this study. Only correlations significant at p = 0.05 are shown; the size of each dot indicates the relative strength of each correlation, whilst colours indicate the direction (blue = positive correlation; red = negative correlation).
(TIFF)

S5 Fig Dendrogram based on an 11 cluster split of the full 46 fatty acid dataset. Membership of each cluster indicated by red boxes.
(TIFF)

S6 Fig Plot indicating the relative stability of differing numbers of clusters, based on adjusted rand criterion. Higher values indicate more stable groupings. Here, 11 groups were chosen as the peak here was the highest until 30+ variables were included, suggesting this was the most parsimonious number of clusters.
(TIFF)

S7 Fig Correlation plot for the reduced set of seven fatty acids following learning vector quantization (LVQ) feature selection and hierarchical LVQ feature selection and ascendant hierarchal clustering applied to the full set of 46 variables. Only correlations significant at p = 0.05 are shown; the size of each dot indicates the relative strength of each correlation, whilst colours indicate the direction (blue = positive correlation; red = negative correlation).
(TIFF)
