# Supplementary material for: Resource polymorphism in European whitefish: Analysis of fatty acid profiles provides more detailed evidence than traditional methods alone
Source: PLoS One. 2019 Aug 20;14(8):e0221338. doi: 10.1371/journal.pone.0221338 (PMC6701781; doi:10.1371/journal.pone.0221338)
Supplement: S1 Table — (DOCX) [file pone.0221338.s001.docx]

S1 Table: Background data on the spatial location, morphometry, physical chemistry and fish fauna of study lakes in the Paatsjoki watercourse.

|  | Lake Vastus | Lake  Paadar | Lake  Muddus | Lake Ukko | Lake  Inari | Lake Vaggatem |
| --- | --- | --- | --- | --- | --- | --- |
| Location | 69^o^03’N,  27^o^07’E | 68^o^52’N,  26^o^35’E | 69^o^00’N,  26^o^50’E | 68^o^58’N,  27^o^40’E | 68^o^58’N,  27^o^40’E | 69^o^13’N,  29^o^11’E |
| Area (km^2^) | 4 | 21 | 48 | 15 | 1043 | 15 |
| Altitude (m a.s.l.) | 146 | 144 | 146 | 119 | 118 | 52 |
| Maximum depth (m) | 15 | 56 | 73 | 60 | 92 | 30 |
| Mean depth (m) | 2.7 | 11.7 | 8.5 | 11.1 | 14.5 | 4 |
| pH | 7.0* | 7.1* | 7.2* | 6.9* | 7.2* | 6.8 |
| Total P (µgl^-1^) | 7* | 7* | 4* | 3* | 5* | 9 |
| Total N (µgl^-1^) | 240* | 160* | 160* | 130* | 150* | 145 |
| Secchi depth (m) | 2 | 6 | 3 | 8 | 6 | 3 |
| Compensation depth (m) | 6 | 5 | 7 | 13 | 8 | 6 |
| Coregonid proportion (%) | 70 | 91 | 86 | 67 | 80 | 78 |
| Fish species and morphs | a,d,e,g,h,i,j,k,l,m | a,b,c,d,e,,g,h,i,j,k,l,m | a,b,c,d,e,f,g,hi,j,k,l,m | a,c,e,f,g,h,i,j,k,l,m,n | a,b,c,d,e,f,g,h,i,j,k,l,m,n,o,p | a,b,c,d,e,g,h,i,j,k,l,n |

Fish species and morphs present within each study lake are indicated by letters: a) LSR whitefish, b) SSR whitefish, c) LDR whitefish, d) DR whitefish, e) brown trout, f) Arctic charr (*Salvelinus alpinus*), g) grayling (*Thymallus thymallus*), h) pike, i) burbot (*Lota lota*), j) perch (*Perca fluviatilis*), k) minnow (*Phoxinus phoxinus*), l) 9-spined stickleback (*Pungitius pungitius*), m) 3-spined stickleback (*Gasterosteus aculeatus*), n) vendace, o) lake trout (*Salvelinus namycush*), p) landlocked salmon (*Salmo salar* m. *sebago*). * Data from Lapland Centre for Economic Development, Transport and the Environment.
