# Supplementary figures and images for: Resource polymorphism in European whitefish: Analysis of fatty acid profiles provides more detailed evidence than traditional methods alone
Source: PLoS One. 2019 Aug 20;14(8):e0221338. doi: 10.1371/journal.pone.0221338 (PMC6701781; doi:10.1371/journal.pone.0221338)

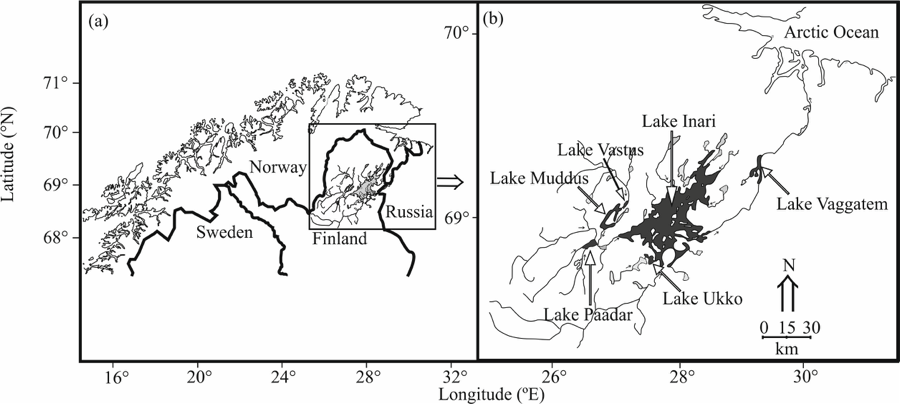

Supplement: S1 Fig — (TIFF) [file pone.0221338.s007.tiff]

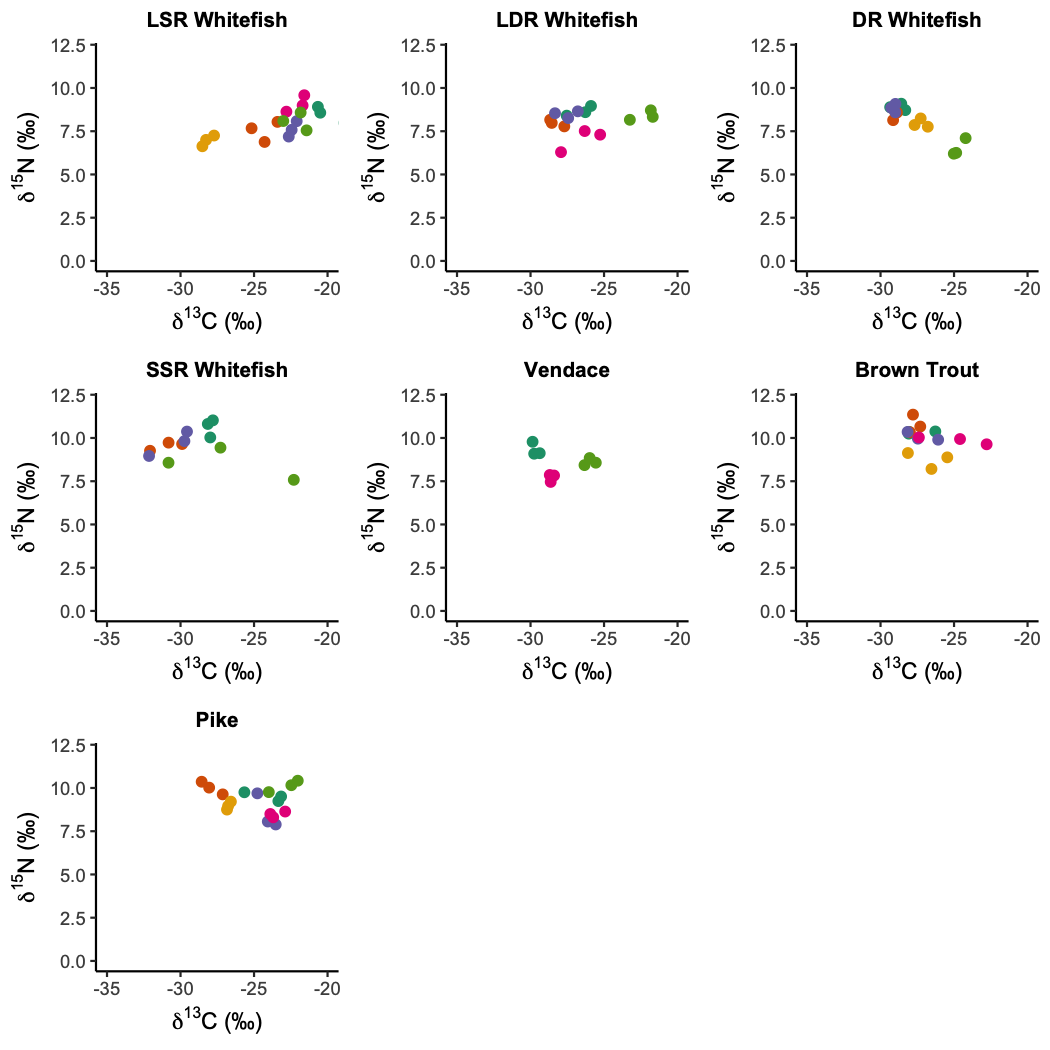

Supplement: S2 Fig — Symbols indicate individual lakes: Lake Inari = dark green circles; Lake Muddus = orange circles; Lake Paadar = purple circles; Lake Ukko = pink circles; Lake Vaggatem = light green circles; Lake Vastus = yellow circles. NB: Not all taxa occur in all lakes. (TIFF) [file pone.0221338.s008.tiff]

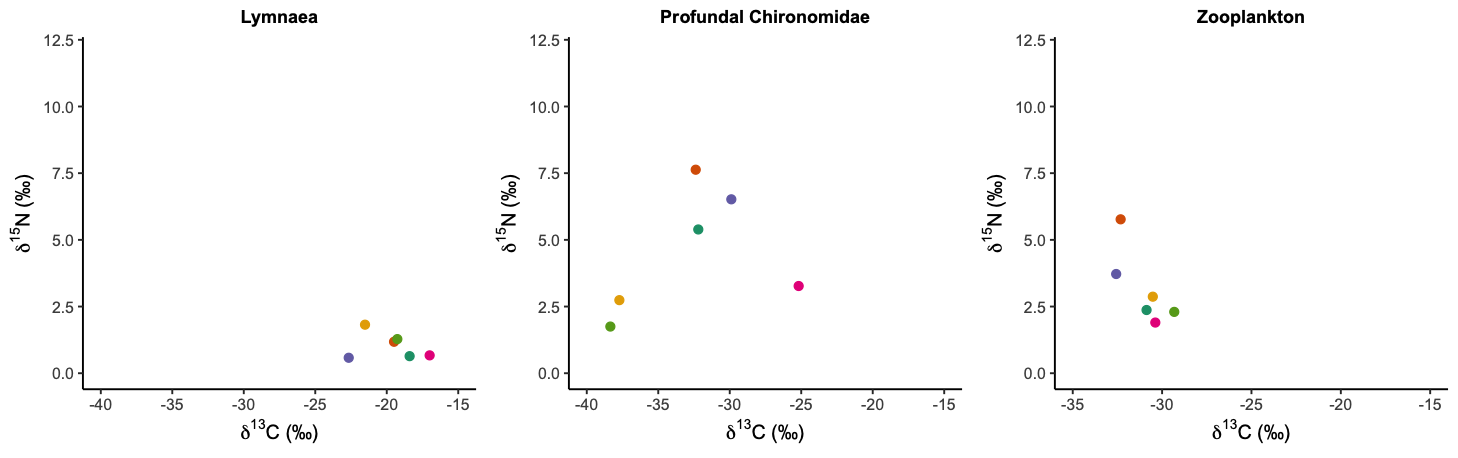

Supplement: S3 Fig — Symbols indicate individual lakes: Lake Inari = dark green circles; Lake Muddus = orange circles; Lake Paadar = purple circles; Lake Ukko = pink circles; Lake Vaggatem = light green circles; Lake Vastus = yellow circles. NB: Not all taxa occur in all lakes. (TIFF) [file pone.0221338.s009.tiff]

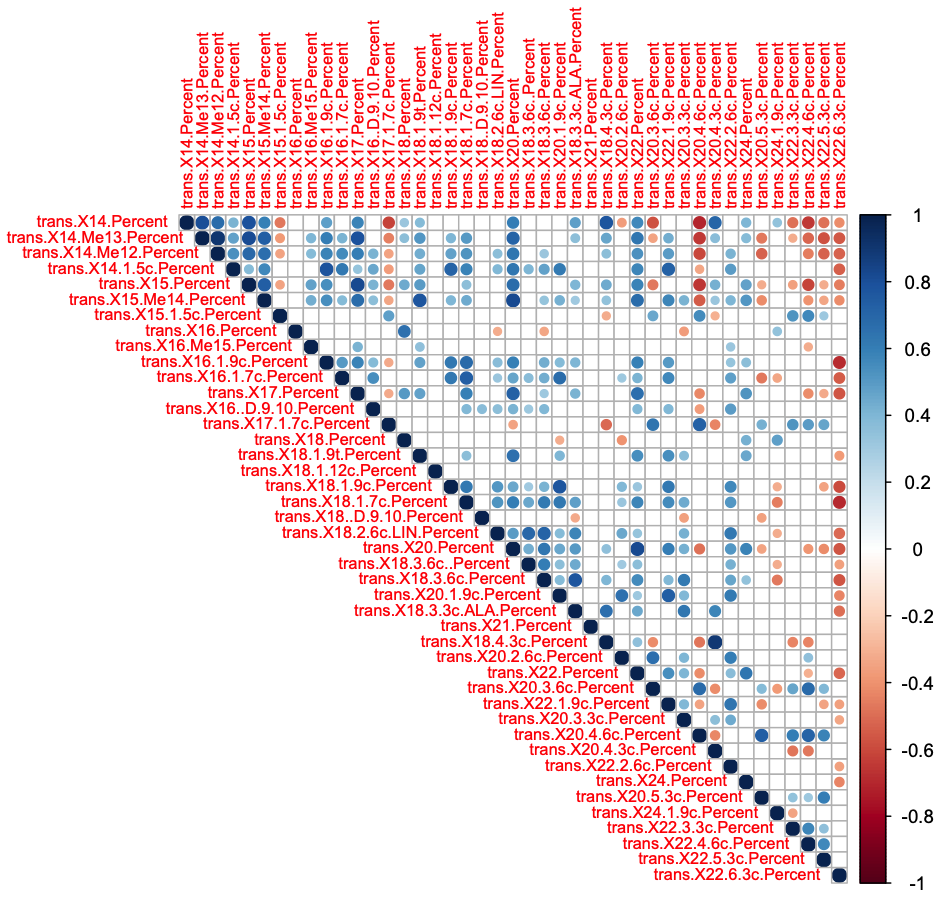

Supplement: S4 Fig — Only correlations significant at p = 0.05 are shown; the size of each dot indicates the relative strength of each correlation, whilst colours indicate the direction (blue = positive correlation; red = negative correlation). (TIFF) [file pone.0221338.s010.tiff]

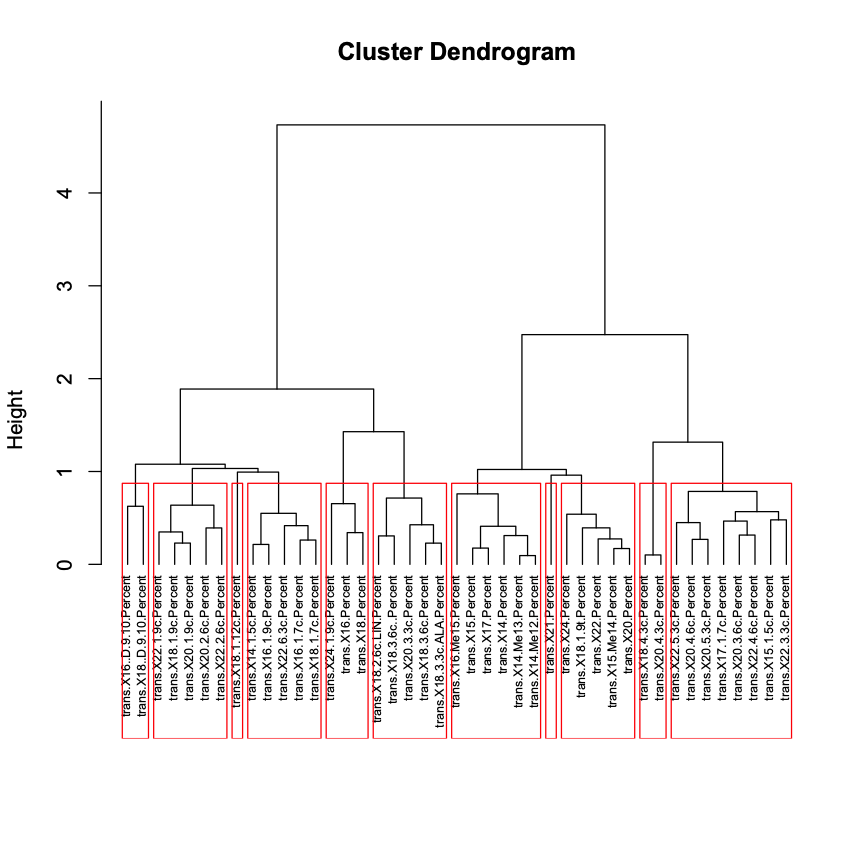

Supplement: S5 Fig — Membership of each cluster indicated by red boxes. (TIFF) [file pone.0221338.s011.tiff]

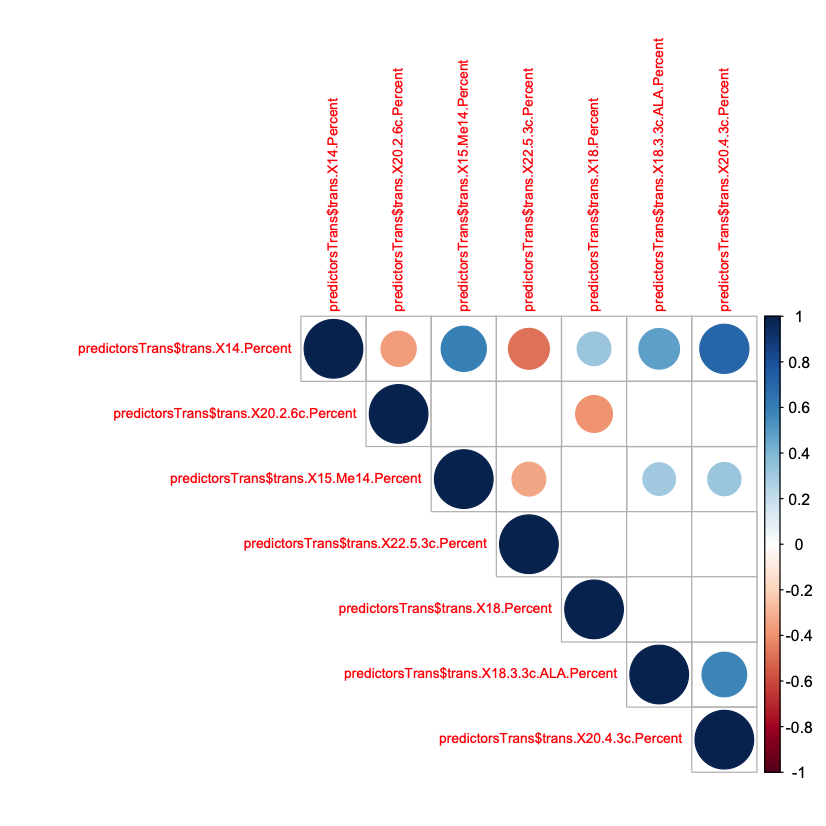

Supplement: S7 Fig — Only correlations significant at p = 0.05 are shown; the size of each dot indicates the relative strength of each correlation, whilst colours indicate the direction (blue = positive correlation; red = negative correlation). (TIFF) [file pone.0221338.s013.tiff]
